# Possible role of microRNA miRNA-IL-25 interaction in mice with ulcerative colitis

**DOI:** 10.1080/21655979.2020.1804176

**Published:** 2020-08-11

**Authors:** Jun Yao, Ruo-yu Gao, Ming-han Luo, Cheng Wei, Ben-hua Wu, Li-liangzi Guo, Li-sheng Wang, Jian-yao Wang, De-feng Li

**Affiliations:** a Department of Gastroenterology, Jinan University of Second Clinical Medical Sciences, Shenzhen People’s Hospital, Shenzhen, Guangdong Province, China; b Department of General Surgery, Shenzhen Children’s Hospital, Shenzhen, Guangdong Province, China

**Keywords:** MicroRNA, ulcerative colitis, miR-135b-5p, miR-691, IL-25

## Abstract

Background: The regulatory network of ulcerative colitis (UC)-associated miRNAs is not fully understood. In this study, we aim to investigate the global profile and regulatory network of UC associated miRNAs in the context of dextran sulfate sodium (DSS). Methods: UC was induced in C57BL mice using DSS. Differentially expressed miRNAs were screened by RNA sequencing and subjected to the Kyoto Encyclopedia of Genes and Genomes Pathway enrichment analysis. RT-qPCR was used to verify the differential expression of miRNAs and candidate target mRNA. Luciferase reporter vector bearing a miRNA binding site was constructed to verify the binding site of the miRNA on mRNA. Results:A total of 95 miRNAs (31 were up-regulated and 64 were down regulated) differentially expressed in the colonic tissues of the UC mice. Among the differentially expressed miRNAs, IL-25 pathway genes were enriched. Subsequent RT-qPCR confirmed a decreased expression of IL-25 and a significant up regulation of IL-25 target miRNAs including mmu-miR-135b-5p, mmu-miR-7239-5p and mmu-miR-691 in UC mice. Conclusion: Using the luciferase assay, we verified the biding sites of mmu-miR-135b-5p and mmu-miR-691 to the IL-25 3ʹUTR. In conclusion, mmu-miR-135b-5p:IL-25 and mmu-miR-691:IL-25 interactions are important pathways that may exert a protective role in UC.

## Graphical abstract

**Figure:**
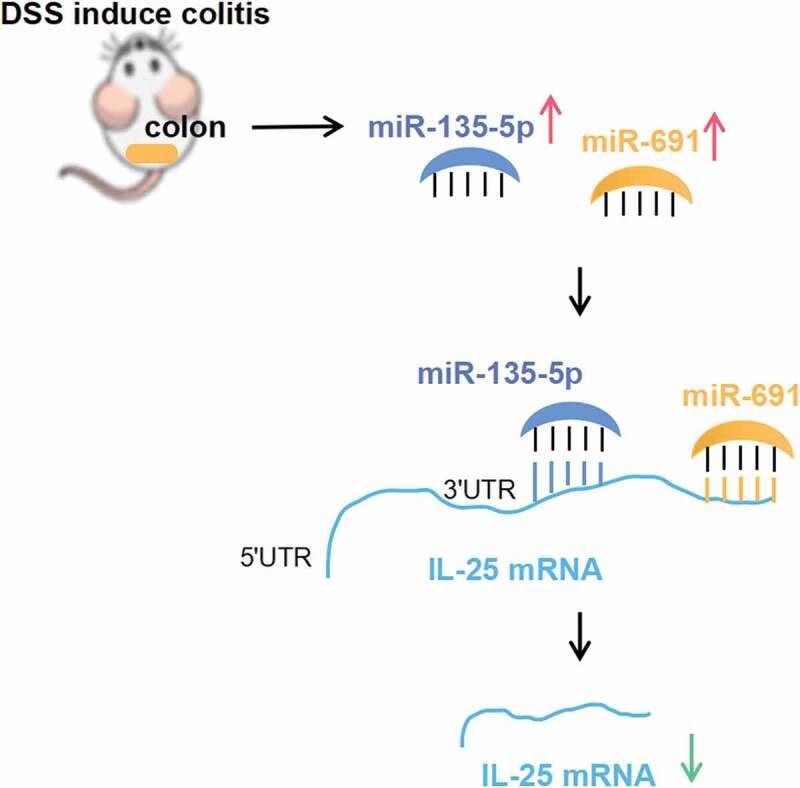

## Introduction

Ulcerative colitis (UC) is an idiopathic chronic inflammatory disease and is one of the two major types of inflammatory bowel disease (IBD). It is estimated that the direct medical cost of UC ranges from $6217 to $11477 (per patient per year) in the United States and €8949 to €10395 in Europe [1]. Although UC is a costly and life-threatening disease for patients with acute severe ulcerative colitis in western countries [1], the pathogenesis of UC remains unknown. Genetic and epigenetic factors are believed to be involved in UC pathogenesis [2].

MicroRNA (miRNA) is an endogenous short (about 21 nt long) non-coding RNA that performs its function primary by binding to the 3ʹUTR of its target mRNA with the seed sequence (5ʹ end 2–8 nt), resulting in targeted mRNA level and protein translational inhibition [3]. Increased evidence suggests that miRNA is one of the important pathogenic factors of inflammatory diseases [4,5]. In UC, miRNAs are dysregulated in human mucosal and epithelial tissues [6–8], playing an important role in the regulation of the intestinal epithelial cell barrier environment and homeostasis, thus regulating inflammation [9,10]. Although studies have revealed miRNA expression profiles in UC patients, including active and/or inactive intestinal mucosa, blood samples [6,11,12], and in special populations, such as a pediatric population [7]. However, most of these studies are based on microarray technology that cannot reflect the global change in UC expression profiling. At the same time, it is difficult to completely control the basic disease context of the recruited patients. Therefore, miRNA profiles based on the experimental model and performed on high-throughput sequencing platform may enhance our understanding of miRNA-mRNA regulatory pathways in UC.

UC is characterized by chronic relapsing intestinal inflammation. Dysregulation in the inflammation-related pathways is a characteristic of this disease. Previously, several studies have suggested inflammation-associated miRNA-mRNA interaction regulatory relationship in UC. For example, microRNA-31 and miR-155 target interleukin 13 receptor α-1 [13]. miR-4284 and miRNA-141 bind directly to the CXCL5 3ʹUTR [7,14]. However, more miRNA/mRNA pathways, especially the regulatory network between UC-related miRNAs and inflammation-related pathways, require investigation.

In this study, we investigated the expressed miRNA profiles using high-throughput sequencing based on a dextran sulfate sodium (DSS)-induced UC mouse model. Next, we analyzed the major enrichment pathways of miRNAs to assess the inflammation-associated miRNAs-mRNA regulatory relationship.

## Materials and methods

### Cell culture

HEK-293 cells (ATCC, Rockville, MD) were cultured in DMEM (Invitrogen, CA, USA) medium containing 10% fetal bovine serum in a 5% CO_2_ incubator at 37°C.

### Establishment of a DSS-induced UC mouse model

UC mouse model was induced via administration of DSS. A total 12 C57BL/6 mice (6–8 weeks old) were randomly resigned to the normal feeding group (NC) or the DSS feeding group (DSS). DSS mice were administrated 2.5% DSS (Biochemicals, CA, USA) in their drinking water for 7 days to induce acute UC. NC mice with regular drinking were used as vehicle controls. The disease activity index(DAI) was scored as per the following scoring system: Weight: 0, no loss; 1, 5%–10% lost; 2, 10%–15% loss; 3, 15%–20% loss; and 4, >20% loss; stool: 0, normal; 2, loose stool; and 4, rib, diarrhea; and bleeding: 0, no blood; 1, presence of blood; 3, obvious blood; and 4, gross blood. DAI and body weight were recorded from day 0 to day 14 after DSS treatment. Spleen weight loss and colon length were determined at day 7 after DSS treatment. To determine the histological change after DSS treatment, the distal colon was fixed in 4% paraformaldehyde to prepare paraffin-embedded blocks on day 7 after DSS treatment. Hematoxylin and eosin (H&E) were used to assess the histological change after DSS treatment. All animal experiments were reviewed and approved by the Jinan University Animal Medical Ethics Committee, China.

### MiRNA sequencing and bioinformatics

Total RNA was isolated from the intestinal tissue of two DSS-induced UC mice and two control mice at day 14 post DSS treatment using TRizol Reagent (Thermo Fisher Scientific, MA, USA) as per the manufactures’ instructions. Total RNA was qualified using Agilent Bioanalyzer 2100 (Agilent, Agilent Technologies, CA, USA). Next, small RNA libraries were generated with a TruSeq Small RNA Sample Preparation Kit (Illumina, CA, USA) as per the manufacture’s recommendation, and the libraries were qualified with Agilent Bioanalyzer 2100. RNA sequencing was performed on an Illumina HiSeq2000 platform (Illumina).

The raw data released from the HiSeq2000 platform were filtered to remove low-quality reads. Reads with ‘adapter-only’ or unclipped were also discarded. The remaining reads were aligned to the mouse NCBI37/mm9 genome sequencing using SOAP V2.0. miRNA was predicted and annotated using NCBI Refseq data and NCBI Genbank data (http://www.ncbi.nih.gov). miRNAs that meet the criteria of fold change ≥ 2 and with a P < 0.01, were identified as differentially expressed miRNAs in UC mice.

To predict potential function of the differentially expressed target gene of miRNAs that may be involved, the Kyoto Encyclopedia of Genes and Genomes (KEGG) pathway analysis was conducted using open-source R package. Q (adjusted P value) < 0.001 was considered to indicate significant enrichment. Candidate miRNA targets were predicted using Targetscan and the interaction network of miRNA-mRNA was visualized by R software.

### Real-time quantitative RT-PCR (RT-qPCR)

The expression of miRNA and mRNA were validated using RT-qPCR. First, total RNA was reverse transcribed using QuantiTect Reverse Transcription Kit (Qiagen, Valencia, CA) and amplified using QuantiTect SYBR Green RT-PCR Kit (Qiagen) as per the manufactures’ instructions. The amplification was performed on an ABI 7500 real-time PCR system (Applied Biosystems, CA, USA). The relative expression of miRNA and mRNA was calculated using the 2^−ΔΔCt^ method. U6 and GAPDH was used as an internal control. Primers used for RT-qPCR are listed in Table 1.

**Table 1.** Primer sequence used in this study.

Primer	Sequence (5ʹ to 3ʹ)
mmu-miR-761	GCAGCAGGGTGAAACTGATCAA
mmu-miR-7002-5p	TTGGCTTCGGGGAGTACTACAT
mmu-miR-505-5p	GGGAGCCAGGAAGTATTGATAGT
mmu-miR-679-3p	AGCAAGGTCCTCCTCACATAGTA
mmu-miR-135b-5p	TATGGCTTTTCATTCCTATGCCGC
mmu-miR-7239-5p	GCCATCCTGACAAAGCTGATG
mmu-miR-691-F	ATTCCTGAAGAGAGGCAGCTGA
IL25-F	CAGCAAAGAGCAAGAACCCC
IL25-R	CCGATTCAAGTCCCTGTCCAA

### Vector construction and transfection

Sequences of IL-25 WT 3ʹUTR, bearing mutant site of mmu-miR-135b-5p or mmu-miR-691 were synthesized and cloned into pmirGL0 (Promega, WI, USA) vector, resulting in Mus-IL-25 wt-pmirGL0, Mus-IL-25Mut-pmirGL0-135b, and Mus-IL-25Mut-pmirGL0-691 vectors. After the vectors were constructed, miRNA mimics, including mmu-miR-135b-5p, mmu-miR-691, and mmu-miRNA NC (negative control), synthesized by GenePharma, Shanghai, China were transfected or co-transfected with 100 nM Mus-IL-25 wt-pmirGL0, Mus-IL-25Mut-pmirGL0-135b, and Mus-IL-25Mut-pmirGL0-691 vectors using Lipofectamine 2000 (Invitrogen, CA, USA) into HEK-293 cells as per the manufacturer’s instructions.

### Luciferase report assay

For luciferase report assay, approximately 8000 cells were seeded on 96-well plates 8 h before the miRNA mimics and vectors transfection. miRNA mimics and vectors were transfected into HEK-293 as mentioned above. After 48 h of culture, luciferase activity was detected usingDual-Glo Luciferase Assay System (Promega, WI, USA) as per the manufacturer’s instruction. The fluorescence values were calculated as firefly fluorescence value/Renilla fluorescence value.

### Statistical analyses

Data are represented as mean ± SD values, unless otherwise mentioned. The differences between the two groups were determined using 2-tailed Student’s t tests. One-way ANOVAs analysis was used for making comparisons among more than three groups with SPSS version 12.0 (SPSS Inc., Chicago, IL, USA). P values < 0.05 were considered statistically significant.

## Results

### Establishment of a DSS-induced UC mice model

**Figure 1.**
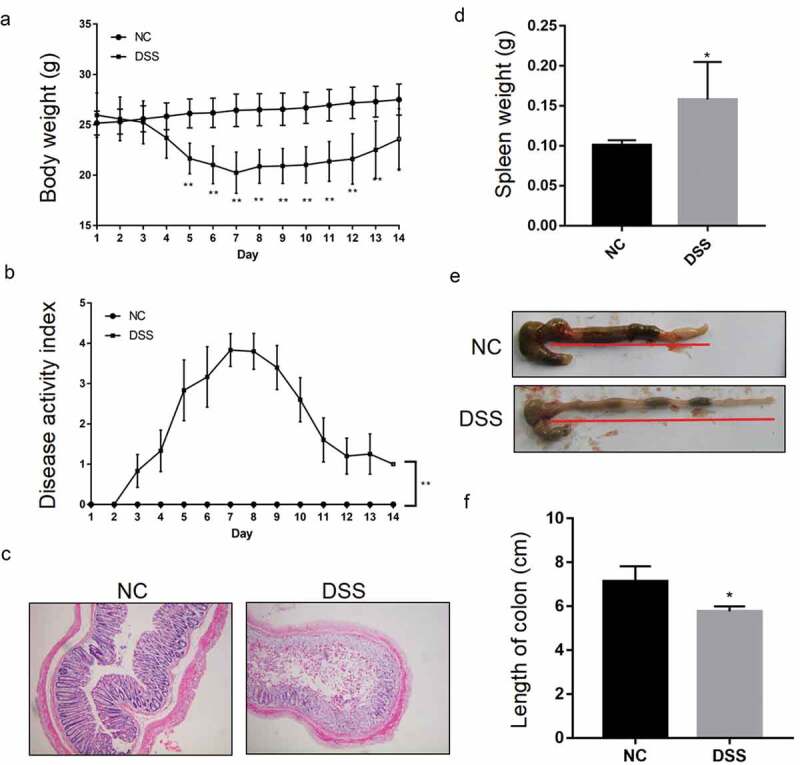
**Successful construction of a DSS-induced UC mice model**. Reduced body weight (a) and increased disease activity index (b) during a 14-day treatment periodin the DSS feeding group (DSS). **P < 0.01 vs. NC (ANOVA). (c) Representative image showing impaired epithelial integrity in the mucosa of DSS mice. Representative image showing increased spleen weight (d) and shortened colon length, indicated by red underline (e, f) in DSS mice compared with the normal feeding group (NC). *P < 0.05 vs. NC (*t* test).

The UC mice model was constructed by adding DSS in the drinking water. After DSS administration, the body weight and DAI of the DSS-induced UC mice and the NC mice were recorded every day for 14 d. As shown in Figure 1, the weight of the NC mice showed no significant changes, while that of the DSS-induced UC mice showed a daily reduction after DSS induction, with the body weight becoming the lowest at 7 d after DSS treatment. After 7 d, the weight of the DSS-induced UC mice gradually recovered to a level similar to that in the NC mice (Figure 1(a)). The DAI of the NC group was 0 across the whole observation period. Compared with the NC group, the DAI of the DSS group increased after DSS treatment and reached the highest value on the 7th day. After 7 d, the value dropped gradually; however, it was still higher than that in the NC group (Figure 1(b)).

After 7 d of DSS treatment, H&E results showed significant epithelial damage in the DSS group, significant loss of crypts, and separation from the muscularis mucosa (Figure 1(c)). In addition, the spleen weight had increased significantly and the colon was significantly shortened compared to that in the NC mice (Figure 1(d–f)). Obvious bloody stools were also observed in the DSS group.

### A wide range of miRNA are differentially expressed in DSS-induced UC mice

**Figure 2.**
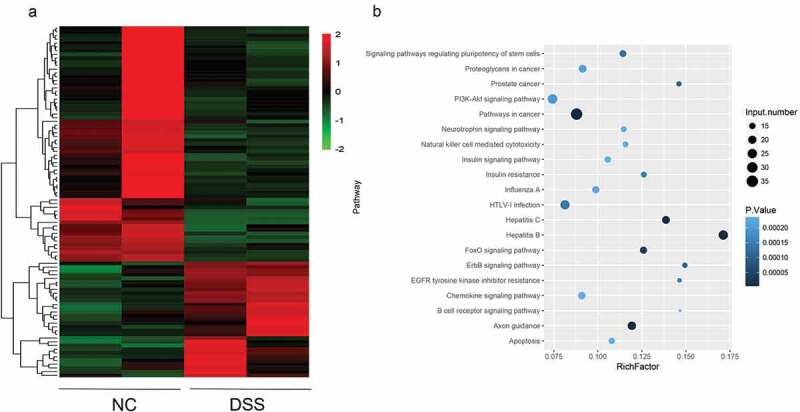
**Differentially expressed miRNAs in UC micescreened by RNA sequencing**. (a) Heat map showing an overview of differentially expressed miRNAs obtained from RNA sequencing data.The red and blue scales represent higher or lower expression levels, respectively. (b) KEGG pathway enrichment analysis showing the potential functions of these differentially expressed miRNAs and the pathways they may possibly be involved in.

Two mice in each group were subjected to miRNA sequencing. The results of miRNA sequencing showed that there were 95 differentially expressed miRNA, of which, 31 were up-regulated and 64 were down regulated. Heatmap revealed that the profile of miRNA expression in DSS-induced UC mice was different from that in the NC mice (Figure 2(a)).

To explore the function of these miRNAs that may be involved, miRNA targets were predicted, and the candidate miRNA targets were subjected to KEGG analysis. The KEGG result showed that these targets were mainly enriched in tumor-associated pathways, such as the pathway in cancer; the PI3K-AKT signaling pathway; immune pathways, such as the chemokine signaling pathway and the apoptosis signaling pathway (Figure 2(b)). Among the up-regulated miRNAs, the up-regulated levels of mmu-miR-135b-5p, mmu-miR-7239-5p, mmu-miR-3070-5p, mmu-miR-1983, and mmu-miR-882 were the highest.

### IL-25 harbors the biding site of the miRNAs that were differentially expressed in UC mice

**Figure 3.**
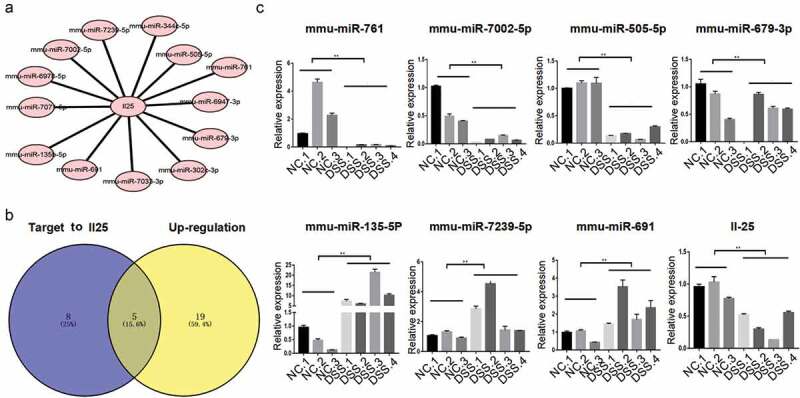
**Bioinformatic and RT-qPCR analyses suggest that miRNAs are differentially expressed in UC mice and targets to IL-25**. (a) Candidate interaction between miRNA and IL-25, a gene belonging to the chemokine pathway, were predicted using Targetscan. (b) Common miRNAs among the up-regulated miRNAs were predicted using RNA-seq and bioinformatic targets of IL-25. (c) RT-qPCR assay differentially validated the expression of IL-25 and its candidate target miRNAs in DSS mice. **P < 0.01 vs. NC (ANOVA).

The target mRNA of the miRNA of the immune-related chemokine signaling pathway was further analyzed. First, the miRNA-mRNA interaction network of the chemokine signaling pathway gene was constructed. In this large interaction network, IL-25 links the largest number of miRNAs. The most 25 up-regulated and down regulated miRNAs in the DSS rice (Table 2) and targeting 13 differentially expressed miRNAs (Figure 3(a)). Among them, 5 miRNAs were up-regulated and 8 were down regulated as per the miRNA sequencing results (Figure 3(b)). Moreover, RT-qPCR showed that mmu-miR-761, mmu-miR-7002-5p, mmu-miR-505-5p, and mmu-miR-679-3p were down regulated in the intestinal tissue of UC, while mmu-miR-135b- 5p, mmu-miR-7239-5p, and mmu-miR-691 were up-regulated (Figure 3(c)). The IL-25 expression was also significantly decreased in the DSS mice.

**Table 2.** The most 25 up/down-regulated miRNAs in the DSS mice.

geneID	NC	DSS	Fold change (DSS/NC)	Up/Down regulation	P value
mmu-miR-135b-5p	2.54	11.15	4.39	Up	0.00
mmu-miR-7239-5p	3.85	13.58	3.53	Up	0.00
mmu-miR-3070-5p	2.95	9.34	3.17	Up	0.01
mmu-miR-1983	8.83	26.98	3.06	Up	0.03
mmu-miR-882	5.87	16.52	2.81	Up	0.02
mmu-miR-7668-3p	12.01	32.26	2.69	Up	0.04
mmu-miR-7661-3p	1.93	5.14	2.66	Up	0.01
mmu-miR-696	1.38	3.59	2.59	Up	0.00
mmu-miR-7084-5p	5.23	13.06	2.50	Up	0.03
mmu-miR-467a-3p	0.75	1.88	2.49	Up	0.01
mmu-let-7 k	4.40	10.90	2.48	Up	0.03
mmu-miR-467 f	0.35	0.85	2.43	Up	0.03
mmu-miR-6978-5p	2.70	6.56	2.43	Up	0.02
mmu-miR-6896-3p	2.42	5.79	2.40	Up	0.01
mmu-miR-466p-3p	0.63	1.50	2.38	Up	0.01
mmu-miR-669 g	0.49	1.17	2.37	Up	0.01
mmu-miR-877-5p	2.56	6.03	2.36	Up	0.02
mmu-miR-7033-3p	1.50	3.50	2.33	Up	0.01
mmu-miR-298-3p	2.63	5.99	2.28	Up	0.02
mmu-miR-34b-3p	1.67	3.75	2.24	Up	0.03
mmu-miR-691	1.40	3.03	2.16	Up	0.02
mmu-miR-3098-5p	1.86	4.01	2.16	Up	0.03
mmu-miR-6985-3p	2.88	6.09	2.11	Up	0.05
mmu-miR-6916-5p	0.90	1.87	2.07	Up	0.03
mmu-miR-761	4.19	1.70	0.41	Down	0.01
mmu-miR-7652-3p	10.49	4.19	0.40	Down	0.03
mmu-miR-302b-5p	8.35	3.27	0.39	Down	0.02
mmu-miR-615-3p	9.50	3.63	0.38	Down	0.02
mmu-miR-1264-3p	3.28	1.25	0.38	Down	0.01
mmu-miR-7228-3p	3.11	1.17	0.38	Down	0.00
mmu-miR-219a-2-3p	9.03	3.10	0.34	Down	0.01
mmu-miR-343	2.24	0.77	0.34	Down	0.00
mmu-miR-7064-5p	1.78	0.61	0.34	Down	0.00
mmu-miR-365-1-5p	4.40	1.49	0.34	Down	0.00
mmu-miR-7002-5p	8.89	2.94	0.33	Down	0.01
mmu-miR-802-5p	6.92	2.19	0.32	Down	0.01
mmu-miR-3080-3p	2.88	0.89	0.31	Down	0.00
mmu-miR-6947-3p	4.22	1.25	0.30	Down	0.00
mmu-miR-6924-3p	2.22	0.65	0.29	Down	0.00
mmu-miR-219b-5p	7.29	2.14	0.29	Down	0.00
mmu-miR-487b-5p	7.56	2.19	0.29	Down	0.00
mmu-miR-6946-5p	5.35	1.53	0.29	Down	0.00
mmu-miR-302 c-5p	1.71	0.48	0.28	Down	0.00
mmu-miR-383-3p	7.55	1.98	0.26	Down	0.00
mmu-miR-505-5p	3.46	0.85	0.25	Down	0.00
mmu-miR-10b-3p	29.01	6.91	0.24	Down	0.00
mmu-miR-10a-5p	17893.46	3872.45	0.22	Down	0.02
mmu-miR-10b-5p	5347.20	1106.07	0.21	Down	0.02
mmu-miR-7674-5p	7.46	1.38	0.18	Down	0.00
mmu-miR-10a-3p	277.22	41.18	0.15	Down	0.00

**Figure 4.**
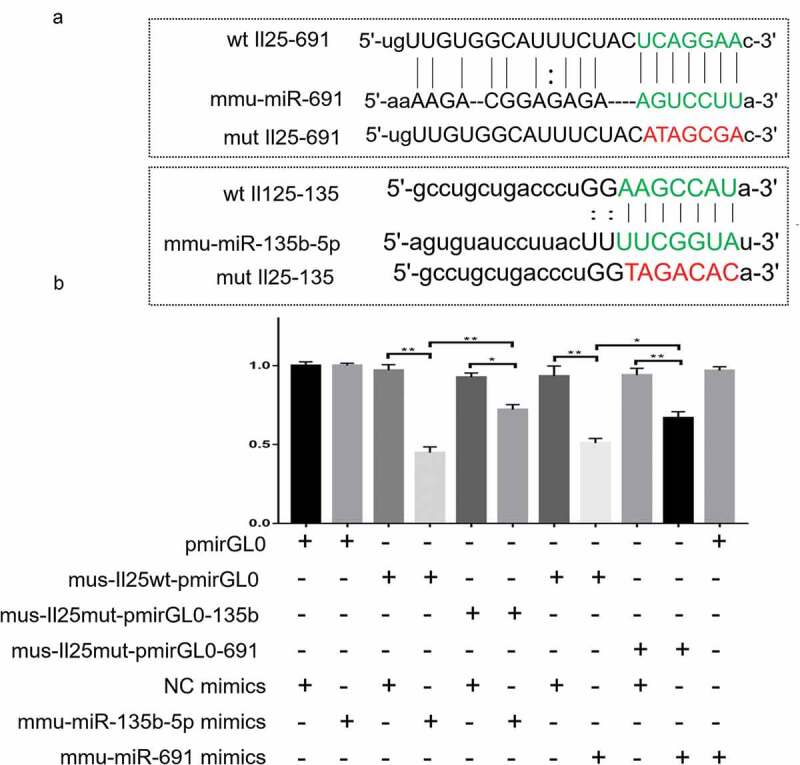
**Luciferase assay showing the IL-25 and miR-691/miR-135b-5p relationship**. (b) Candidate binding sites of IL-25 and miR-691/miR-135b-5p predicted by Targetscan. (c) Luciferase report system verified the binding site of mmu-miR-691:IL-25 and mmu-miR-13b-5p:IL-25.

The bioinformatics analyses suggested that there are binding sites between the up-regulated miRNAs and IL-25; therefore, we constructed a 3ʹUTR luciferase reporter vector containing the IL-25 wild and mutant sequences (Figure 4(a)) and co-transfected with miRNA mimics to HEK293 cells to validate the potential binding sites for IL-25 and miRNA. As shown in Figure 4, the luciferase activity of mmu-miR-135b-5p mimics+mus-IL-25 wt-pmirGL0 was significantly reduced compared to that of the NC mimics and mus-IL-25 wt-pmirGL0 co-transfection group. After IL-25 mutation, there was no significant change in the luciferase activity in the mus-IL-25mut-pmirGL0-135b+NC mimics group, while the luciferase activity of mus-IL-25mut-pmirGL0-135b+mmu-miR-135b-5p mimics cells was obviously restored. The same trend was also observed in the mmu-miR-691 group. The luciferase activity of mmu-miR-691 mimics+mus-IL-25 wt-pmirGL0 was lower than that of NC mimics+mus-IL-25 wt-pmirGL0, while the luciferase activity of mmu-miR-691 mimics+mus-IL-25mut-pmirGL0-691 was significantly increased.

## Discussion

In this study, we showed 94 differentially expressed miRNAs by comparing DSS-induced miRNA expression profiles in UC mice and normal mice. Of these, 31 were up-regulated and 64 were down regulated. The KEGG pathway enrichment analyses revealed that these differentially expressed miRNAs were mainly enriched in pathways such as those involved in tumor, proliferation, inflammation, and apoptosis. In particular, we analyzed the miRNA regulatory network in the chemokine signaling pathway and found that IL-25 regulates the largest number of bound miRNAs. Further, RT-qPCR results verified the downregulation of IL-25 and several target miRNAs of IL-25 in the intestinal tissue of UC mice; the expressions of mmu-miR-7002-5p, mmu-miR-505-5p, and mmu-miR-679-3p were down regulated, while those of mmu-miR-135b-5p, mmu-miR-7239-5p and mmu-miR-691 were up-regulated. Finally, we propose a regulatory relationship between mmu-miR-135b-5p: IL-25 and mmu-miR-691: IL-25.

Throughput sequencing revealed a series of differentially expressed miRNAs in UC. It is noteworthy that differentially expressed miRNAs are mainly enriched in tumors; PI3K-AKT signaling pathways; and inflammatory pathways, such as the chemokine signaling pathway and apoptotic pathways. Among them, the PI3K-AKT signaling pathway is involved in the regulation of apoptosis, inflammation, immunity, and barrier function in UC [15,16]. A previous web-based database study that compared the differences in the gene expressions of UC and CD found that chemokine receptor 7 (CCR7) and IL-8 of the chemokine pathway occupy important sites in the regulation of UC network. This finding suggests an important role of the chemokine signaling pathway in UC [17]. Apoptosis frequently occurring in the epithelial cells and lymphocytes is a common cell biological event that contributes to disrupted barrier function and UC development [18–22]. Our sequencing results also showed that DSS-induced UC differentially expressed miRNAs that may be involved in the regulation of apoptotic behavior.

UC is characterized by chronic relapsing intestinal inflammation. Therefore, we focus on the miRNAs involved in the chemokine signaling pathway. In the chemokine pathway, IL-25 is the most miRNA-targeted gene, suggesting its potential regulatory function in UC. IL-25 is a Th2-related factor that promotes the production of Th2 cytokines including IL-4, IL-13, and TGF-β1 to alternatively activated macrophages [23]. It has been validated as downregulated in both sera and inflamed mucosa in patients with active IBD [24]. In the DSS-induced UC mouse model, IL-25 is significantly downregulated and plays a role in inhibiting inflammatory factor IFN-γ and promoting the expression of anti-inflammatory factor IL-10 [25]. Another animal study shows that IL-25 can prevent intestinal inflammation [26]. Intraperitoneal injection of recombinant IL-25 has shown to relieve DAI, limit pathological changes, and prolong survival time in an acute UC mouse model [27]. These studies indicate that IL-25 plays the role of defending against the formation and progress of UC. However, the regulatory mechanism of IL-25 is not fully understood. In the current study, RT-qPCR assay confirmed the target miRNAs of IL-25, mmu-miR-761, mmu-miR-7002-5p, mmu-miR-505-5p, and mmu-miR-679-3p were down regulated, while those of mmu-miR-135b-5p, mmu-miR-7239-5p, and mmu-miR-691 were up-regulated in the intestinal tissues of UC mice. IL-25 was down regulated. This is consistent with the sequencing results. Mechanistically, miRNA acts as an mRNA level inhibitor by targeting the 3ʹUTR of mRNA. Therefore, we focused on the up-regulated miRNAs and verified the biding of miRNA and IL-25 using luciferase assay. Finally, we confirmed the relationship of mmu-miR-691: IL-25 and mmu-miR-135b-5p: IL-25. Although the function of mmu-miR-691 remains unclear, studies have suggested its up regulation upon PM2.5 intratracheal instillation and virus infection in mice, indicating its involvement in immune defense [28,29]. Another target of IL-25, mmu-miR-135b-5p, has been shown to be up-regulated in UC and IBD-associated tumor tissues [30–32] and is associated with colorectal cancer development and progression [33,34]. Currently, the exact role of mmu-miR-691 and mmu-miR-135b-5p in UC remains unknown. Our study confirms the interaction between mmu-miR-691/mmu-miR-135b-5p and Il25, indicating the role of mmu-miR-691 and mmu-miR-135b-5p in protecting against gut inflammation in the context of DSS-induced colitis.

## Conclusions

In sum, we revealed the miRNA profile of UC in DSS-induced UC mice using high-throughput sequencing. Moreover, we predicted the inflammation-related miRNA-IL-25 network and verified the regulatory relationship of mmu-miR-691:IL-25 and mmu-miR-13b-5p:IL-25. Current knowledge of miRNA-mRNA interaction in UC is limited. Our findings support that IL-25 is an important node of the UC inflammatory pathway, and this especially highlights the fact that the mmu-miR-691:IL-25 and mmu-miR-13b-5p:IL-25 networks have the potential to protect against UC, which may provide a clue for UC pathogen and targeted therapy analysis.

## Highlights

1. A series UC associated miRNAs are found in DSS-induce mice model.
2. IL-25 is a key target gene of UC associated miRNAs.
3. mmu-miR-135b-5p: IL-25 mmu-miR-691:IL-25 interactions are important pathways of UC development.
